# The Efficacy and Clinical Safety of Various Analgesic Combinations for Post-Operative Pain after Third Molar Surgery: A Systematic Review and Meta-Analysis

**DOI:** 10.1371/journal.pone.0127611

**Published:** 2015-06-08

**Authors:** Alvin Ho Yeung Au, Siu Wai Choi, Chi Wai Cheung, Yiu Yan Leung

**Affiliations:** 1 Oral and Maxillofacial Surgery, Faculty of Dentistry, The University of Hong Kong, Hong Kong Special Administrative Region, People Republic of China; 2 Laboratory and Clinical Research Institute for Pain, Department of Anaesthesiology, Faculty of Medicine, The University of Hong Kong, Hong Kong Special Administrative Region, People Republic of China; University of Bari, ITALY

## Abstract

**Objectives:**

To run a systematic review and meta-analysis of randomized clinical trials aiming to answer the clinical question “which analgesic combination and dosage is potentially the most effective and safe for acute post-operative pain control after third molar surgery?”.

**Materials and Methods:**

A systematic search of computer databases and journals was performed. The search and the evaluations of articles were performed by 2 independent reviewers in 3 rounds. Randomized clinical trials related to analgesic combinations for acute post-operative pain control after lower third molar surgery that matched the selection criteria were evaluated to enter in the final review.

**Results:**

Fourteen studies with 3521 subjects, with 10 groups (17 dosages) of analgesic combinations were included in the final review. The analgesic efficacy were presented by the objective pain measurements including sum of pain intensity at 6 hours (SPID6) and total pain relief at 6 hours (TOTPAR6). The SPID6 scores and TOTPAR6 scores of the reported analgesic combinations were ranged from 1.46 to 6.44 and 3.24 – 10.3, respectively. Ibuprofen 400mg with oxycodone HCL 5mg had superior efficacy (SPID6: 6.44, TOTPAR6: 9.31). Nausea was the most common adverse effect, with prevalence ranging from 0-55%. Ibuprofen 200mg with caffeine 100mg or 200mg had a reasonable analgesic effect with fewer side effects.

**Conclusion:**

This systematic review and meta-analysis may help clinicians in their choices of prescribing an analgesic combination for acute post-operative pain control after lower third molar surgery. It was found in this systematic review Ibuprofen 400mg combined with oxycodone HCL 5mg has superior analgesic efficacy when compared to the other analgesic combinations included in this study.

## Introduction

The combination of analgesics from different classes may provide additive analgesic effects with lesser side effects than when a single drug is used [1, 2]. It has also been suggested that the combination of analgesics can provide different mechanisms of action and therefore multimodal coverage of a wider spectrum of pain, thus enable the drug components to provide potential synergistic effect [3]. Moreover, in terms of safety, analgesic combinations may allow a lower dose of single drug component, which may result in a lower incidence of adverse effects. As multiple pathways of human body’s nociception were suggested [4], analgesic combinations are appropriate for pain management and has been recommended by World Health Organization (WHO) [5], the American Pain Society (APS) [6] and the American College of Rheumatology (ACR) [7].

Acute post-operative dental pain model has been suggested as an excellent analgesic model because the pain can be debilitating [8]. Wisdom tooth surgery has been commonly used in studies to investigate the efficacy of single or combination analgesics [9–11]. Review of the literature demonstrated that analgesic combinations of NSAIDs or acetaminophen and opioids were superior to single drug in terms of analgesic effect and/ or side-effect for acute post-operative pain [12].

However, among the enormous number of possible combinations, there is a lack of knowledge regarding which therapeutic analgesic combination and the respective drug dosages is more superior in terms of analgesic efficacy and its clinical safety. Therefore the purpose of the study was to conduct a systematic review and meta-analysis to answer a clinical question “which analgesic combination and dosage is potentially the most effective and safe for acute post-operative pain control after third molar surgery?”

## Materials and Methods

A systematic review and meta-analysis was designed to perform two rounds of comprehensive searches of literature with relevance to the clinical question and a round of critical evaluation to identify relevant articles that could be included in the final review. Two authors (A.H.Y. and L.Y.Y.) were judges in the two rounds of search and the evaluation round, and worked independently according to the protocol and were blinded to each other’s choice. Disagreements between two judges were discussed and solved by consent. A third party (N.S.) was consulted if any consensus to a disagreement could not be reached.

### First round search

Three databases (Pubmed, Embase and the Cochrane Library) were searched. There were no restriction criteria set on language or publication date. The last date of the search was 1st March, 2013. The electronic search was performed using the keywords:

1. third molar;
2. wisdom tooth;
3. dentoalveolar surgery
4. analgesic
5. painkiller
6. acute dental pain
7. combination

A search was performed by (1 or 2 or 3) AND (4 or 5 or 6 or 7)

A list of articles with the search strategy was generated. Each abstract of the articles was reviewed. The full articles were retrieved if there was inadequate information in the abstracts or the abstracts were missing. Articles relevant to the study of analgesics combination and third molar surgery were selected and included in the next round.

### Second round search and selection

To expand the search for possible articles that were relevant to the topic, a manual search was performed on three international oral and maxillofacial surgery journals (Oral Surgery, Oral Medicine, Oral Pathology, Oral Radiology, and Endodontology; International Journal of Oral and Maxillofacial Surgery; and Journal of Oral and Maxillofacial Surgery). The manual search of these 3 journals was limited to the period from March 2003 to Feb 2013. Articles relevant to the treatment of post-operative dental pain and uses of combination analgesia were selected in this round.

A reference list search was performed from the manual search articles and the selected articles of the first round articles. Articles that were relevant to the study of post-operative dental pain and combination of analgesic efficacy and clinical safety were selected.

In addition to the articles from the first round, all articles were evaluated. Two independent judges (A.H.Y and L.Y.Y) carried out independent selection of the articles entered this round, according to the following selection criteria:

1. Articles must be limited to **third molar surgery**;
2. Articles must be limited for assessing the **combination of analgesic**;
3. Articles must be randomized clinical trials.

Articles had to fulfill all these three criteria to be selected to enter the third round evaluation.

### Third round evaluation

Articles entering the third round were evaluated by two independent judges (A.H.Y and L.Y.Y) based on the following criteria.

1. The study must contain one or more of the following information about the patients who underwent wisdom tooth surgery: basic demographic data (mean age, gender of patients); number of wisdom teeth removed; and baseline pain intensity.
2. The study must contain a placebo group.
3. The articles must consist of the following two objective pain measurements that were commonly used in analgesic studies:
  - Sum of pain intensity difference in 6 hours (SPID6);
  - Total pain relief in 6 hours (TOTPAR6).
4. The articles must include the adequate description on the side effect, patient tolerability and safety of the drugs.

Articles had to fulfill all criteria to be included in the final review. Table 1 summarized the eligibility criteria for articles included in the final review. Table 2 listed the excluded studies and the reasons for exclusion.

**Table 1:** Eligibility criteria for articles included in the final review.

Type of studies: Randomized control trial with a minimal sample size of 15 patients and placebo.
Type of participants: Patients had surgical extraction of 1 or more third molar which was partial or complete bony impacted mandibular third molar; experience moderate or severe pain associated with the procedure.
Type of intervention: For the acute post-operative pain control, patients received any combination analgesia, the type of drugs combination and the route and the dosage of therapy must be clearly reported.
Type of outcome measures:
Primary outcome
• Sum of Pain Intensity at 6 hours (SPID6)Total pain relief at 6 hours (TOTPAR6)
Secondary outcome
• To report different drug combination adverse effect
The method, criteria and time of evaluation must be clearly reported. The analgesic efficiency outcomes specific for SPID6 and TOTPAR6 needs to be reported.

**Table 2:** Excluded studies at the third round and explanation for exclusion.

Authors	Title	Reasons for exclusion
1. Sveen K, et al [71].	Paracetamol/codeine in relieving pain following removal of impacted mandibular third molars.	No SPID6 and TOTPAR6 data
2. Hellem S, et al [72].	A model for evaluating the analgesic effect of a new fixed ratio combination analgesic in patients undergoing oral surgery.	No SPID6 and TOTPAR6 data
3. Forbes JA, et al [73].	An evaluation of the analgesic efficacy of three opioid-analgesic combinations in postoperative oral surgery pain.	No SPID6 and TOTPAR6 data
4. Quiding H, et al [74].	An analgesic study with repeated doses of phenazone, phenazone plus dextropropoxyphene, and paracetamol, using a visual analogue scale.	No SPID6 and TOTPAR6 data No placebo
5. Quiding H, et al [75].	Paracetamol plus supplementary doses of codeine. An analgesic study of repeated doses.	No placebo
6. Edmondson HD, et al [76].	Analgesia following oral surgery: a comparative study of Solpadeine and a soluble form of dextropropoxyphene napsylate and paracetamol.	No SPID6 and TOTPAR6 dataNo placebo
7. Laska EM, et al [77].	Effect of caffeine on acetaminophen analgesia.	No placebo
8. Dionne RA, et al [78].	Comparison of conorphone, a mixed agonist-antagonist analgesic, to codeine for postoperative dental pain.	No placebo
9. Ahlstrom U, et al [79].	Multiple doses of paracetamol plus codeine taken immediately after oral surgery.	No SPID6 and TOTPAR6 data
10. Dahl E, et al [80].	Acetylsalicylic acid compared with acetylsalicylic acid plus codeine as postoperative analgesics after removal of impacted mandibular third molars.	No placebo
11. Rosen M, et al [81].	Suprofen compared to dextropropoxyphene hydrochloride and paracetamol (Cosalgesic) after extraction of wisdom teeth under general anaesthesia.	No placebo
12. Frame JW, et al [82].	A double-blind placebo-controlled comparison of three ibuprofen/codeine combinations and aspirin.	No SPID6 and TOTPAR6 data
13. Happonen RP, et al [83].	A combination of acetylsalicylic acid and codeine phosphate versus acetylsalicylic acid as postoperative analgesics after mandibular third molar surgery.	No SPID6 and TOTPAR6 data
14. Hill CM, et al [84].	Ibuprofen given pre- and post-operatively for the relief of pain.	No SPID6 and TOTPAR6 data
15. Liashek P, et al [85].	Effect of pretreatment with acetaminophen-propoxyphene for oral surgery pain.	No SPID6 and TOTPAR6 data
16. Sagne S, et al [86].	Analgesic efficacy and side-effect profile of paracetamol/codeine and paracetamol/dextropropoxyphene after surgical removal of a lower wisdom tooth.	No placebo
17. Dupuis R, et al [87].	Preoperative Flurbiprofen in Oral Surgery: A Method of Choice in Controlling Postoperative Pain.	No SPID6 and TOTPAR6 data
18. McQuay HJ, et al [88].	Codeine 20 mg increases pain relief from ibuprofen 400 mg after third molar surgery. A repeat-dosing comparison of ibuprofen and an ibuprofen-codeine combination.	No placebo
19. Becker J, et al [89].	Double blind biometric study on postoperative effects of analgesics.	No placebo
20. Giglio JA, et al [90].	Double-blind comparison of meclofenamate sodium plus codeine, meclofenamate sodium, codeine, and placebo for relief of pain following surgical removal of third molars.	No SPID6 and TOTPAR6 data
21. Habib S JA, et al [91].	A study of the comparative efficacy of four common analgesics in the control of postsurgical dental pain.	No placebo
22. Walton GM, Rood JP [92].	A comparison of ibuprofen and ibuprofen-codeine combination in the relief of post-operative oral surgery pain.	No placebo
23. Skoglund LA JA, et al [93].	Analgesic efficacy of acetaminophen 1000 mg, acetaminophen 2000 mg, and the combination of acetaminophen 1000 mg and codeine phosphate 60 mg versus placebo in acute postoperative pain.	No SPID6 and TOTPAR6 data
24. Hellman M JA, et al [94].	Analgesic efficacy of an ibuprofen-codeine combination in patients with pain after removal of lower third molars.	No placebo
25. Lownie JF JA, et al [95].	Comparison of the safety and efficacy of a combination analgesic Myprodol and Ponstan in the treatment of dental pain.	No placebo
26. Lysell L JA, et al [96].	Pain control after third molar surgery--a comparative study of ibuprofen (Ibumetin) and a paracetamol/codeine combination (Citodon).	No placebo
27. McQuay HJ JA, et al [97].	A multiple dose comparison of combinations of ibuprofen and codeine and paracetamol, codeine and caffeine after third molar surgery.	No placebo
28. Dolci G JA, et al [98].	Analgesic efficacy and the tolerance for piroxicam-beta-cyclodextrin compared to piroxicam, paracetamol and placebo in the treatment of postextraction dental pain.	No SPID6 and TOTPAR6 data
29. Petersen JK JA, et al [99].	The effect of an ibuprofen-codeine combination for the treatment of patients with pain after removal of lower third molars.	No placebo
30. Dionne RA, et al [100].	Analgesic efficacy of flurbiprofen in comparison with acetaminophen, acetaminophen plus codeine, and placebo after impacted third molar removal.	No SPID6 and TOTPAR6 data
31. Berge TI [101].	Pattern of self-administered paracetamol and codeine analgesic consumption after mandibular third-molar surgery.	No placebo
32. McGurk M, et al [102].	Clinical comparison of dexketoprofen trometamol, ketoprofen, and placebo in postoperative dental pain.	No SPID6 and TOTPAR6 data
33. Merry AF, et al [103].	Tenoxicam and paracetamol-codeine combination after oral surgery: a prospective, randomized, double-blind, placebo-controlled study.	No SPID6 and TOTPAR6 data
34. Breivik EK, et al [104].	Combining diclofenac with acetaminophen or acetaminophen-codeine after oral surgery: a randomized, double-blind single-dose study.	No placebo
35. Dionne RA, et al [105].	Additive analgesic effects of oxycodone and ibuprofen in the oral surgery model.	No placebo
36. Caruso FS, et al [106].	MorphiDex pharmacokinetic studies and single-dose analgesic efficacy studies in patients with postoperative pain.	No SPID6 and TOTPAR6 data
37. Ziccardi VB, et al [107].	Single-dose vicoprofen compared with acetaminophen with codeine and placebo in patients with acute postoperative pain after third molar extractions.	No SPID6 and TOTPAR6 data
38. Medve RA, et al [108].	Tramadol and acetaminophen tablets for dental pain.	No SPID6 and TOTPAR6 data
39. Comfort MB, et al [109].	A study of the comparative efficacy of three common analgesics in the control of pain after third molar surgery under local anaesthesia.	No placebo
40. Daniels SE, et al [110].	The analgesic efficacy of valdecoxib vs. oxycodone/acetaminophen after oral surgery.	No SPID6 and TOTPAR6 data
41. Fricke JR, et al [111].	A double-blind, single-dose comparison of the analgesic efficacy of tramadol/acetaminophen combination tablets, hydrocodone/acetaminophen combination tablets, and placebo after oral surgery.	No SPID6 and TOTPAR6 data
42. Garibaldi JA, et al [112].	Evaluation of ketorolac (Toradol) with varying amounts of codeine for postoperative extraction pain control.	No placebo
43. James Fricke TV, et al [113].	Rofecoxib compared to oxycodone/acetaminophen for post-operative dental pain.	2004 Merck & co voluntary worldwide withdrawal rofecoxib from the market due to risks of MI, Stroke, CVD
44. Kiersch TA, et al [114].	The onset of action and the analgesic efficacy of Saridon (a propyphenazone/paracetamol/ caffeine combination) in comparison with paracetamol, ibuprofen, aspirin and placebo (pooled statistical analysis).	No SPID6 and TOTPAR6 data
45. Macleod AG, et al [115].	Paracetamol Versus Paracetamol-Codeine in the Treatment of Post-Operative Dental Pain: A Randomized, Double-Blind, Prospective Trial.	No placebo
46. Joshi A, et al [116].	A double-blind randomised controlled clinical trial of the effect of preoperative ibuprofen, diclofenac, paracetamol with codeine and placebo tablets for relief of postoperative pain after removal of impacted third molars.	No SPID6 and TOTPAR6 data
47. Jung YS, et al [117].	Onset of analgesia and analgesic efficacy of tramadol/acetaminophen and codeine/acetaminophen/ibuprofen in acute postoperative pain: a single-center, single-dose, randomized, active-controlled, parallel-group study in a dental surgery pain model.	No SPID6 and TOTPAR6 result not available
48. Korn S, et al [118].	Comparison of rofecoxib and oxycodone plus acetaminophen in the treatment of acute pain: a randomized, double-blind, placebo-controlled study in patients with moderate to severe postoperative pain in the third molar extraction model.	2004 Merck & co voluntary worldwide withdrawal rofecoxib from the market due to risks of MI, Stroke, CVD
49. Chang DJ, et al [119].	Analgesic efficacy of rofecoxib compared with codeine/acetaminophen using a model of acute dental pain.	2004 Merck & co voluntary worldwide withdrawal rofecoxib from the market due to risks of MI, Stroke, CVD
50. Barroso AB, et al [120].	Efficacy and safety of combined piroxicam, dexamethasone, orphenadrine, and cyanocobalamin treatment in mandibular molar surgery.	No placebo
51. Haglund B, et al [121].	Combining paracetamol with a selective cyclooxygenase-2 inhibitor for acute pain relief after third molar surgery: a randomized, double-blind, placebo-controlled study.	No SPID6 and TOTPAR6 data
52. Desjardins PJ, et al [122].	A double-blind randomized controlled trial of rofecoxib and multidose oxycodone/acetaminophen in dental impaction pain.	2004 Merck & co voluntary worldwide withdrawal rofecoxib from the market due to risks of MI, Stroke, CVD
53. Leone M, et al [123].	Comparison of methylprednisolone and ketoprofen after multiple third molar extraction: a randomized controlled study.	No placebo
54. Borel JF, et al [124].	Treating pain after dental surgery: a randomised, controlled, double-blind trial to assess a new formulation of paracetamol, opium powder and caffeine versus tramadol or placebo.	No SPID6 and TOTPAR6 data
55. Merry A, et al [125].	Combined acetaminophen and ibuprofen for pain relief after oral surgery in adults: a randomized controlled trial.	No SPID6 and TOTPAR6 data No placebo
56. Daniels SE, et al [126].	Evaluation of the dose range of etoricoxib in an acute pain setting using the postoperative dental pain model.	No SPID6 and TOTPAR6 data
57. Isiordia- Espinoza MA, et al [127].	Preemptive analgesic effectiveness of oral ketorolac plus local tramadol after impacted mandibular third molar surgery.	No SPID6 and TOTPAR6 data No placebo
58. Brown JD, et al [128].	Evaluation of Multiday Analgesia With Etoricoxib in a Double-blind, Randomized Controlled Trial Using the Postoperative Third-molar Extraction Dental Pain Model.	No SPID6 and TOTPAR6 data

### Final review

Articles entering the final review were being assessed of the efficacies and the adverse effects of the analgesic combinations reported in the studies. Drug efficacy was reported by the two objective pain measurements, SPID6 and TOTPAR6 (See **Objective pain measurements**). Single drug analgesics reported in the included studies were not assessed. The reported SPID6 and TOTPAR6 value of the placebos in the included articles reported and compared with the analgesic combinations. When there were multiple studies reporting the same analgesic combination and same dosage, the mean SPID6 and mean TOTPAR6 of the analgesic combination and the respective placebo was calculated according to the formula:

MeanSPID6/TOTPAR6ofananalgesiccombinationorplacebo=[(SPID6orTOTPAR6instudyA)×(numberofsubjectsofstudyA)+(SPID6orTOTPAR6instudyB)×(numberofsubjectsofstudyB)+…+(SPID6orTOTPAR6instudyX)×(numberofsubjectsofstudyX)]/Totalnumberofsubjectsinthestudies.

The adjusted effect of an analgesic combination reported in each included study was calculated by offsetting the placebo effect within the same study to report its actual effect. The formulae to calculate the adjusted SPID6 and TOTPAR6 were as follows:

1. Adjusted SPID6 = SPID6 of drug—SPID6 of placebo
2. Adjusted TOTPAR6 = TOTPAR6 of drug—TOTPAR6 of placebo

The adverse effects of the analgesic combinations of the included studies were reported. The proportion of subjects complaining of an adverse effect of the analgesic combination were reported and compared. When there were multiple studies reporting the same analgesic combination of the same dosage, the mean proportion of subjects presenting with the adverse effect were reported.

### Objective pain measurements

1 **Sum of pain intensity difference in 6 hours (SPID6)**. SPID6 measured the difference of the sum of pain intensity score in the first 6 hours post-operatively. Pain intensity score was reported subjectively by the subject on a four-point scale (0 = none; 1 = slight; 2 = moderate; 3 = severe). The baseline pain intensity score was recorded after third molar surgery and local anaesthesic effect was subsided. Analgesics were administered afterwards.

The Pain Intensity Difference (PID) was measured by the pain intensity score at baseline minus the pain intensity score at a given observation time point, which was recorded hourly in the first 6 hours. The sum of Pain Intensity Difference (SPID) for the 0 to 6-hour observation period was reported as SPID6. The higher score represents more effective analgesia.

2 **Total pain relief in 6 hours (TOTPAR6)**. Pain relief was measured by a categorical rating scale (0 = none; 1 = slight; 2 = moderate; 3 = good; 4 = complete). Pain relief after analgesic consumption was recorded at different post-operative time points, which was recorded hourly in the first 6 hours. The summation of pain relief score on each hour in the first 6 hours resulted in TOTPAR 6. It was defined as the area under the curve of the pain relief scores against the corresponding time interval. For example if a patient had complete pain relief immediately after taken the analgesic, and sustained it for the full 6 hours of observation period, the maximum TOTPAR6 would be (6 hours x 4) 24. The higher the score represented the analgesic was more effective.

### Data Synthesis

Meta-analysis of the data was performed by first looking at the heterogeneity of the studies included in this paper. Cochrane Q and quantification of dispersion between studies using I^2^, values were calculated using Comprehensive Meta-Analysis (version 2.2.064, Englewood, New Jersey). The random effects model was chosen and significance level was set at 0.05.

The manuscript was based on a master thesis of the first author, which was submitted in September 2013 to the University of Hong Kong. The meta-analysis of the study was performed by the second author. A re-search was performed in September 2014 using the keywords and found no new studies to be included. The authors therefore prefer to use the original search date for the systematic review and meta-analysis.

## Results

**Fig 1:**
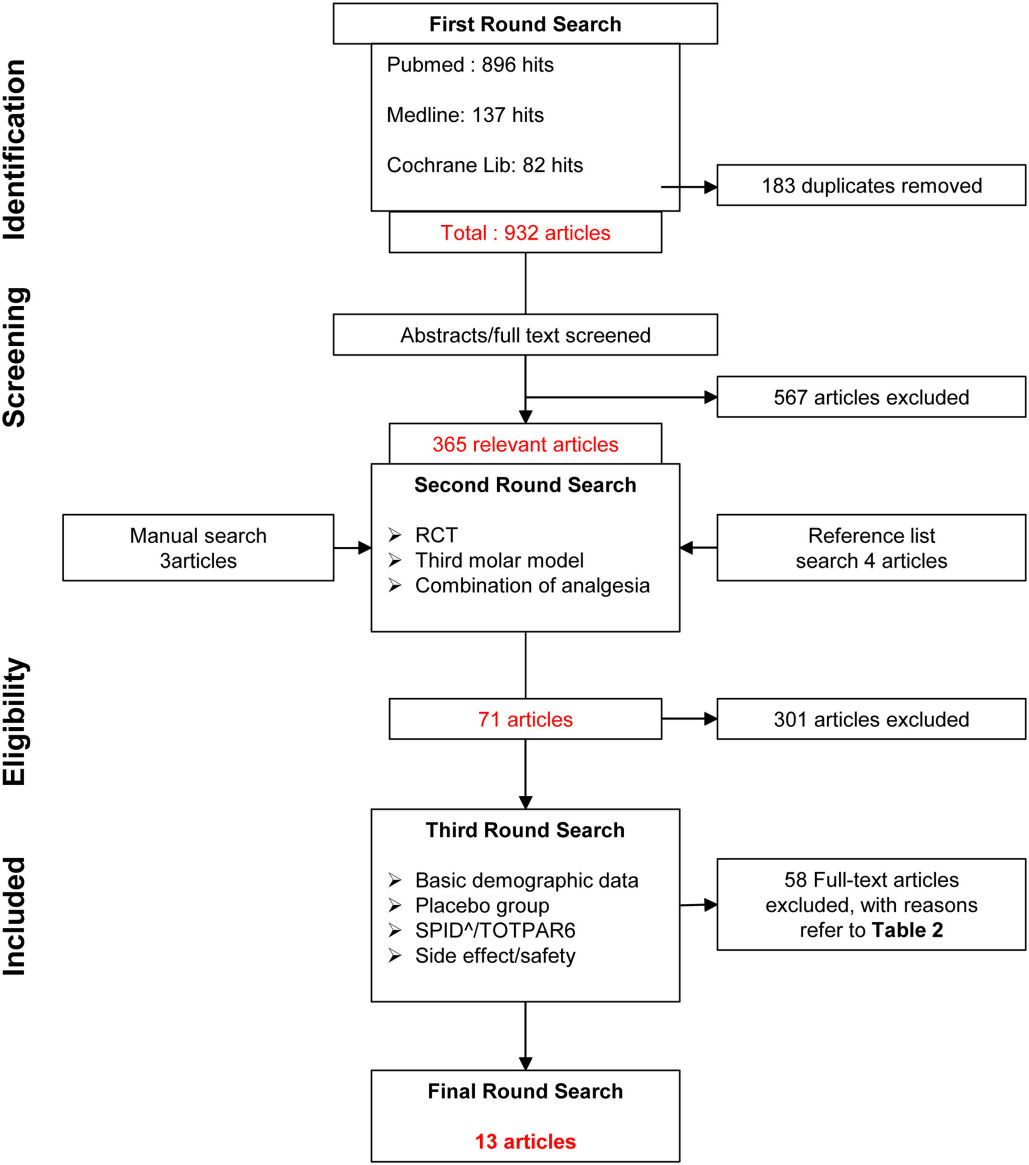
Flow diagram for article selection.

A flow diagram of the three rounds of search and evaluation was presented in Fig 1. The first round search of the computer base, covering the period from the earliest available date to 1^st^ March 2013, yielded 896 hits from PubMed, 137 hits from Medline and 82 hits from the Cochrane Library. One hundred and eighty three hits were duplications and were removed. The abstracts of 932 articles were screened, 365 articles were considered relevant to the study of the efficacy and clinical safety of analgesic combination of post-operative acute dental pain, with 567 articles were considered irrelevant and were excluded. The second round search yielded 4 additional articles from manual search and 4 additional articles from reference search. After selection, 71 articles met the three criteria and entered the third round for evaluation. Fifty-eight studies failed to meet one or more of the criteria in the evaluation round and were excluded. Fourteen studies fulfilled the eligibility criteria and entered the final review.

### General findings

The 14 included studies [13–25] of the final review were all randomized clinical trial with placebo control. These articles were published between 1986 and 2012. Two were multi-center studies [21, 25], thirteen were double blinded single oral dose studies [13, 14, 16–25] and one study was double blinded with repeated doses [15]. All studies reported the basic demographic data.

There were a total of 3521 subjects included the 14 included studies. Of these, 1748 subjects received different combinations and dosages of analgesics and 629 subjects received placebo. The remaining 1144 subjects of these studies had single drug analgesics and were excluded from the review.

Ten groups of drug combinations were identified in the final review according to the type of drug combined. They were 1) acetaminophen + codeine phosphate; 2) acetaminophen + hydrocodone bitartrate; 3) acetaminophen + oxycodone HCL; 4) acetaminophen + ibuprofen 5) aspirin + caffeine; 6) aspirin + codeine phosphate; 7) aspirin + caffeine + butalbital + codeine phosphate; 8) ibuprofen + oxycodone HCL; and 9) ibuprofen + caffeine; 10) ibuprofen + codeine phosphate

There were 17 drug combinations with different dosages and were listed below.

1. Acetaminophen 650mg + codeine phosphate 60mg
2. Acetaminophen 600mg + codeine phosphate 60mg
3. Acetaminophen 300mg + codeine phosphate 30mg
4. Acetaminophen 1g + codeine phosphate 30mg
5. Acetaminophen 1g + hydrocodone bitartrate 10mg
6. Acetaminophen 500mg + hydrocodone bitartrate 7.5mg
7. Acetaminophen 325mg + oxycodone HCL 5mg
8. Acetaminophen 500mg + ibuprofen 200mg
9. Acetaminophen 1g + ibuprofen 400mg
10. Aspirin 650mg + caffeine 65mg
11. Aspirin 650mg + codeine phosphate 60mg
12. Aspirin 325mg + caffeine 40mg + butalbital 50mg + codeine phosphate 15mg
13. Ibuprofen 400mg + oxycodone HCL 5mg
14. Ibuprofen 200mg + caffeine 200mg
15. Ibuprofen 200mg + caffeine 100mg
16. Ibuprofen 200mg + caffeine 50mg
17. Ibuprofen 400mg + codeine phosphate 25.6mg

### Efficacy of analgesic combinations

The efficacies of the analgesic combinations in terms of SPID6 and TOTPAR6 were reported in Table 3.

**Table 3:** Summary of efficacy of various analgesic combinations of the included studies.

Drug Combinations	Study	SPID6 (placebo)	Adjusted SPID6	TOTPAR6 (Placebo)	Adjusted TOTPAR6
Acetaminophen 650mg + codeine phosphate 60mg	Sunshine [24] (n = 31)	4.66 (1.65)	3.01	13.37 (8.3)	5.07
Acetaminophen 600mg + codeine phosphate 60mg	Cooper [13] (n = 31)	5.26(1.75)		11.97 (6.25)	
	Forbes [16] (n = 27)	3.48 (0.25)		8.19 (2.91)	
	Forbes [16] (n = 17)	4.65 (0.13)		10.53 (2.00)	
	*Calculated Mean*	4.48 (0.84)	3.64	10.28 (4.08)	6.20
Acetaminophen 300mg + codeine phosphate 30mg	Forbes [73] (n = 93)	2.78 (0.51)		6.61 (3.35)	
	Forbes [73] (n = 43)	3.12 (0.37)		7.44 (2.37)	
	Gatoulis [20] (n = 119)	4.14 (3.79)		6.09 (3.54)	
	*Calculated Mean*	3.47 (2.01)	1.46	6.51 (3.27)	3.24
Acetaminophen 1g + codeine phosphate 30mg	Daniels [129] (n = 113)	1.00 (0.14)	0.86	1.87 (0.44)	1.43
Acetaminophen 1g + hydrocodone bitartrate 10mg	Fricke [19] (n = 65)	3.7 (0)	3.70	10.3 (3.1)	7.20
Acetaminophen 500mg + hydrocodone bitartrate 7.5mg	Forbes [14] (n = 94)	3.57 (0.51)		8.66 (3.35)	
	Litkowski [21] (n = 62)	3.32 (0.69)		8.36 (5.05)	
	*Calculated Mean*	3.47 (0.58)	2.89	8.54 (4.03)	4.51
Acetaminophen 325mg + oxycodone HCL 5mg	Litkowski [21] (n = 62)	3.58 (0.69)	2.89	9.53 (5.05)	4.48
Acetaminophen 500mg + ibuprofen 200mg	Daniels [129] (n = 173)	1.30 (0.14)	1.16	2.36 (0.44)	1.92
Acetaminophen 1g + ibuprofen 400mg	Daniels [129] (n = 168)	1.47 (0.14)	1.33	2.58 (0.44)	2.14
Aspirin 650mg + caffeine 65mg	Forbes [17] (n = 66)	2.88 (0.12)	2.76	6.8 (1.99)	4.81
Aspirin 650mg + codeine phosphate 60mg	Moore [23] (n = 38)	2.2 (0.4)	1.80	6.9 (2.5)	4.40
Aspirin 325mg+caffeine 40mg + butalbital 50mg + codeine phosphate 15mg	Forbes [18] (n = 41)	3.46 (0.37)	3.09	9.07 (2.37)	6.70
Ibuprofen 400mg + oxycodone HCL 5mg	Litkowski [21] (n = 62)	7.78 (0.69)		14.98 (5.05)	
	Van Dyke [25] (n = 186)	6.54 (0.32)		13.3 (4.2)	
	Calculated Mean	6.85 (0.41)	6.44	13.72 (4.41)	9.31
Ibuprofen 200mg + caffeine 200mg	McQuay [97] (n = 29)	3.5 (0)	3.50	9.5 (0)	9.50
Ibuprofen 200mg + caffeine 100mg	McQuay [97] (n = 30)	3.1 (0)	3.10	10.3 (0)	10.30
Ibuprofen 200mg + caffeine 50mg	McQuay [97] (n = 30)	1.5 (0)	1.50	7.0 (0)	7.00
Ibuprofen 400mg + codeine phosphate 25.6mg	Daniels [129] (n = 169)	1.23 (0.14)	1.09	2.23 (0.44)	1.79

Seven of the 17 different analgesic combinations with different dosages described in the included studies involved acetaminophen combining with an opioid (codeine phosphate, hydrocodone bitartrate or oxycodone HCL), with the adjusted SPID6 and adjusted TOTPAR6 of ranged from 1.46–3.7 and 3.24–7.2 respectively. Four of these combinations involved different dosages of acetaminophen combined with codeine phosphate. There was no obvious difference of efficacy in terms of SPID6 and TOTPAR6 between acetaminophen 650mg + codeine phosphate 60mg and acetaminophen 600mg + codeine phosphate 60mg. But these two combinations were more effective than acetaminophen 300mg + codeine phosphate 30mg, with adjusted SPID6 and adjusted TOTPAR6 at least 2.1 and 1.6 times higher, respectively, than the lower dosage combination.

Three of the analgesic combinations from the included studies involved aspirin as a major analgesic component, with the adjusted SPID6 and adjusted TOTPAR6 were ranged from 1.8–3.09 and 4.4–6.7, respectively. The four drugs combination of aspirin 325mg+caffeine 40mg + butalbital 50mg + codeine phosphate 15mg showed the highest efficacy in terms of adjusted SPID6 (3.09) and TOTPAR6 (6.7) among the three, followed by aspirin 650mg + caffeine 65mg and aspirin 650mg + codeine phosphate 60mg.

Seven of the analgesic combinations reported in the included studies had ibuprofen as a major analgesic component, with the adjusted SPID6 and adjusted TOTPAR6 were ranged from 1.5–6.44 and 7.0–10.3, respectively. Ibuprofen 400mg + oxycodone HCL 5mg showed the highest adjusted SPID6 then the other three combinations of ibuprofen with caffeine in different dosages, which was at least 1.84 times better in adjusted SPID6. For the analgesic combinations of ibuprofen with caffeine, it seemed that the analgesic efficacy did not drastically increase when the dosage of caffeine was increase from 100mg to 200mg, which was interpreted by similar adjusted SPID6 and TOTPAR6 findings. However, ibuprofen 200mg + caffeine 50mg was obviously less effective when compared to the two combinations of ibuprofen 200mg and caffeine in higher dosages.

Among the 17 different analgesic combinations reported in the included studies, ibuprofen 400mg + oxycodone HCL 5mg had the highest adjusted SPID6 (6.44), and a very higher adjusted TOTPAR6 (9.31), representing its efficacy could be the superior than the other different analgesic combinations reported in this study.

### Meta-analysis and Forest plots

**Fig 2:**
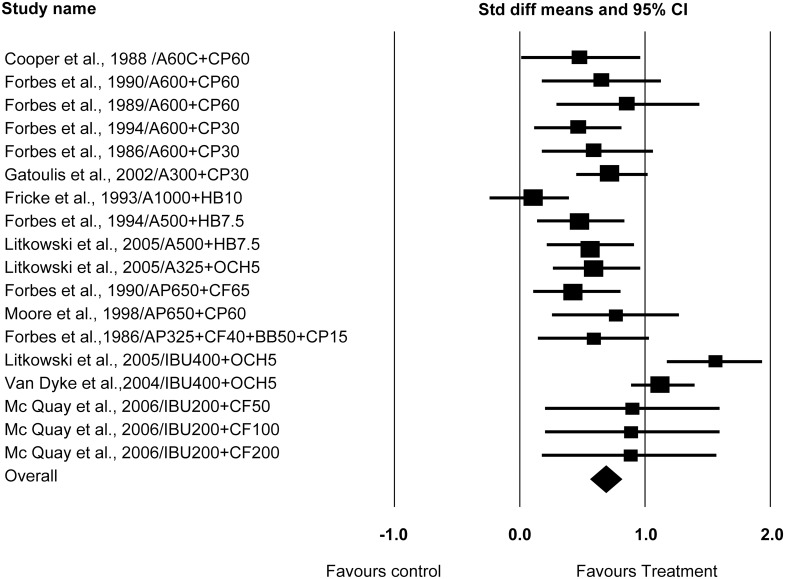
Forest plot according to SPID6.

**Fig 3:**
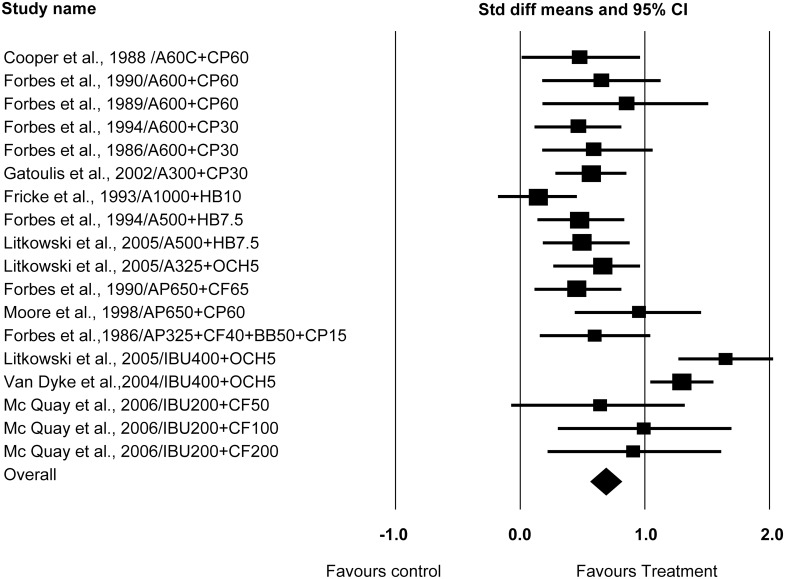
Forest plot according to TOTPAR.

Studies were analyzed separately both according to the SPID6, and then according to the TOTPAR6 scores obtained. The observed between study dispersion, (Cochrane Q value) calculated according to SPID6 and TOTPAR were both p<0.0001, with 17 degrees of freedom (18 studies being included in this analysis) which shows homogenous treatment according to the random effects model. The I^2^ value calculated according to SPID6 and TOTPAR was both 0.0%, which represents less than moderate heterogeneity. Forest plots were presented according to either SPID6, or TOTPAR (Figs 2 and 3). Both figures confirmed all analgesic combinations were better than the placebo, and showed Ibuprofen 400mg with oxycodone 5mg offered the highest analgesic effect after lower third molar surgery.

### Safety of analgesic combinations

The summary of the adverse effects of the various analgesic combinations from the included studies was listed in Table 4. The common adverse effects were mostly related to the side effects of opioids, including drowsiness, dizziness, headache and nausea. Nausea was the most common adverse effect in opioids containing combinations, ranging from 2.4% to 55%. Other rarer adverse effects (e.g. leg numbness, chills, itchy, dry mouth, sweating) were also reported in most of the analgesic combinations but in general the prevalence was low.

**Table 4:** Summary of reported adverse effects of various analgesic combinations of the included studies. (N.R. = Not Reported).

	Drowsi-ness	Dizzi-ness	Ear-ache	Head-ache	Nausea	Numbness in legs	Chills	Itching	Dry mouth	Hot feeling	Red face and neck	Sweat-ing	Restless-ness	Tired-ness	Rash
Acetaminophen 650mg + codeine phosphate 60mg (n = 31) [24]	9.70%	N.R.	N.R.	9.70%	9.70%	N.R.	N.R.	N.R.	N.R.	N.R.	N.R.	N.R.	N.R.	N.R.	N.R.
Acetaminophen 600mg + codeine phosphate 60mg Total n = 48 [15, 16]	6.25%	4.16%	N.R.	N.R.	10.40%	N.R.	N.R.	N.R.	N.R.	N.R.	N.R.	N.R.	N.R.	N.R.	N.R.
Acetaminophen 300mg + codeine phosphate 30mg Total n = 274 [15, 16, 20, 130, 131]	1.46%	2.12%	N.R.	0.70%	11%	0.36%	N.R.	N.R.	0.70%	N.R.	N.R.	N.R.	N.R.	N.R.	0.36%
Acetaminophen 1g + codeine phosphate 30mg (n = 113) [129]	N.R.	12.40%	N.R.	18.60%	32.70%	N.R.	N.R.	N.R.	N.R.	N.R.	N.R.	N.R.	N.R.	N.R.	N.R.
Acetaminophen 1g + hydrocodone bitartrate 10mg (n = 67) [19]	10.50%	22.40%	N.R.	15%	55%	N.R.	N.R.	N.R.	N.R.	N.R.	N.R.	N.R.	N.R.	N.R.	N.R.
Acetaminophen 500mg + hydrocodone bitartrate 7.5mg Total n = 177 [14, 21, 130]	8.47%	6.20%	N.R.	0.56%	15.25%	N.R.	N.R.	0.56%	N.R.	N.R.	N.R.	N.R.	N.R.	N.R.	N.R.
Acetaminophen 325mg + oxycodone HCL 5mg (n = 61) [21]	N.R.	4.90%	N.R.	3.30%	41%	N.R.	N.R.	N.R.	N.R.	N.R.	N.R.	N.R.	N.R.	N.R.	N.R.
Acetaminophen 500mg + ibuprofen 200mg (n = 173) [129]	N.R.	6.90%	N.R.	11.00%	24.90%	N.R.	N.R.	N.R.	N.R.	N.R.	N.R.	N.R.	N.R.	N.R.	N.R.
Acetaminophen 1g + ibuprofen 400mg (n = 168) [129]	N.R.	8.90%	N.R.	11.30%	19.60%	N.R.	N.R.	N.R.	N.R.	N.R.	N.R.	N.R.	N.R.	N.R.	N.R.
Aspirin 650mg+ caffeine 65mg (n = 78) [17]	N.R.	2.56%	N.R.	1.30%	2.56%	1.28%	2.60%	N.R.	N.R.	1.28%	1.30%	2.60%	N.R.	N.R.	N.R.
Aspirin 650mg + codeine phosphate 60mg (n = 39) [23]	9.80%	4.90%	N.R.	22%	19.50%	N.R.	N.R.	N.R.	N.R.	N.R.	N.R.	N.R.	N.R.	N.R.	N.R.
Aspirin 325mg+caffeine 40mg + butalbital 50mg + codeine phosphate 15mg (n = 48) [18]	N.R.	6.25%	2.00%	2.00%	2.01%	N.R.	N.R.	N.R.	N.R.	2.01%	N.R.	2.01%	2.01%	2.01%	N.R.
Ibuprofen 400mg + oxycodone HCL 5mg Total n = 249 [21, 25]	13.25%	1.20%	N.R.	0.40%	2.40%	N.R.	N.R.	N.R.	N.R.	N.R.	N.R.	N.R.	N.R.	N.R.	N.R.
Ibuprofen 200mg + caffeine 200mg (n = 29)[22]	N.R.	N.R.	N.R.	N.R.	N.R.	N.R.	N.R.	N.R.	N.R.	N.R.	N.R.	N.R.	N.R.	N.R.	N.R.
Ibuprofen 200mg + caffeine 100mg (n = 30)[22]	N.R.	3.30%	N.R.	3.30%	N.R.	N.R.	N.R.	N.R.	N.R.	N.R.	N.R.	N.R.	N.R.	N.R.	N.R.
Ibuprofen 200mg + Caffeine 50mg (n = 30)[22]	N.R.	N.R.	N.R.	N.R.	N.R.	N.R.	3.30%	N.R.	N.R.	N.R.	N.R.	N.R.	N.R.	N.R.	N.R.
Ibuprofen 400mg + codeine phosphate 25.6mg (n = 169) [129]	N.R.	12.40%	N.R.	18.60%	32.70%	N.R.	N.R.	N.R.	N.R.	N.R.	N.R.	N.R.	N.R.	N.R.	N.R.

Among the 7 combinations of acetaminophen and an opioids (codeine phosphate, hydrocodone bitartrate or oxycodone HCL), it was noted acetaminophen 1g + hydrocodone bitartrate 10mg had the highest incidence of adverse effects, with 55% of the subjects complaining of nausea or vomiting, 22.4% of the subjects with dizziness, 15% with headache and 10.5% with drowsiness. Acetaminophen 500mg + hydrocodone bitartrate 7.5mg was reported to have fewer subjects with adverse effect, which was likely to be related to the reduced dosage of hydrocodone bitartrate, with only 15.25% of the subjects experienced nausea and 8.47% with drowsiness. There were also 41% of the subjects who took the combination of acetaminophen 325mg and oxycodone HCL 5mg experienced nausea. The combinations of acetaminophen and codeine phosphate had fewer adverse effects reported when compared to the combinations of acetaminophen and hydrocodone bitartrate or oxycodone HCL. From the included studies, it seemed that reduced dosages of acetaminophen and codeine phosphate did not result in a reduced incidence of the side effects of the opioids.

The adverse effects of the two studies reported the use of ibuprofen 400mg + oxycodone HCL 5mg were pooled. The prevalence of the subjects who experienced drowsiness was 13.25%. The other adverse effects were related to the side effect of the opioid oxycodone HCL but the prevalences were low (0.4–2.4%).

In contrast to the analgesic combinations containing opioids, combinations of an NSAID with caffeine were reported to have much fewer adverse effects. In the same study reporting combinations of ibuprofen 200mg and 3 different dosages of caffeine, there seemed to have no obvious difference in terms of prevalence of adverse effect with the increased dosage (up to caffeine 200mg).

## Discussion

Systematic reviews and meta-analyses have become increasingly popular in medicine [26, 27]. It helps clinicians to keep up-to-date clinical practice guideline and facilitate researchers to use them as a starting point for new guideline formation and future research [28, 29]. It can also provide a high-level overview of a particular research or clinical question by the process of identify, select, synthesize and appraise all high quality research evidence [30]. According to the oxford levels of evidence, systematic review of randomized controlled trials (RCT) is considerate to be level 1 evidence [27]. This study tried to summarize objectively the efficacy and clinical safety of various analgesic combination of post-operative acute dental pain. There were only randomized controlled trials (RCT) with placebo were selected into the final round for analysis, the protocol was straightly followed the PRISMA statement (Preferred Reporting Items for Systematic reviews and Meta-Analyses) [31] and it helps to ensure the clarity and transparency of the systematic reviews conducted.

Post-operative pain after third molar surgery has become a frequently used model in the studies of acute pain clinical trials. This is because third molar surgery is one of the commonest procedure with sufficient numbers of patients to make studies relatively easy to perform [32]. It is also a sensitive method for demonstrating the efficacy of oral analgesic agents [19]. It is because the patient sample in dental pain model is homogenous in pain stimulus, and the post-operative pain is frequently moderate or severe in intensity. Moreover, absence of multiple surgical complication factors comparing to other major surgical procedures, trauma, or other pain stimuli reduced to variables of the surgical procedure and outcome. In addition, third molar surgical procedures can be easily categorized, and the obtained data in dental pain model can substantiate the assay sensitivity of the clinical trials, and therefore it is useful in predicting the general analgesic efficacy of NSAIDs [33, 34].

Pooling of data in a systematic review were occasionally criticized to be “mixing apples and oranges”, especially if there were obvious heterogeneity of the included studies. In this study, the heterogeneity of the included studies in the Final Review has been tested to be less than moderate (I^2^ = 0.0%), which enabled a representable meta-analysis to be performed.

The measurement of analgesic efficacy is usually performed by comparing patient’s subjective evaluation of pain before and after administration of the analgesics [35]. For a long time, SPID and TOTPAR are the most commonly used methods to measure the efficacy of an analgesic and were well validated [36–38]. They were used in the research context for comparisons between the efficacies of different analgesics [39], and now routinely used in analgesic studies [40–42]. In this systematic review, formulae of adjusted SPID and adjusted TOTPAR were developed by correction of the placebo effect of the respective studies of the analgesics. This may allow direct cross-studies comparison of the analgesic combination efficacies and to reduce the heterogeneity of the placebo effects in different studies.

Analgesic combinations have been proved to be more effective in pain control when compared to single drug [43, 44]. The concurrent use of ibuprofen and paracetamol was the most widely studied analgesic combination. It was shown in a Cochrane Review that 400mg ibuprofen / 1000mg paracetamol combination has superior analgesic effect when compared to ibuprofen or paracetamol alone or the combination of the two of lower dosage [44]. However, there were no other meta-analysis in the literature comparing the effectiveness and side effects of different analgesic combinations.

This systematic review and meta-analysis of analgesic combinations reported the objective analgesic efficacy and the adverse effects of various analgesic combinations studied in the literature. One of the key findings of this study was ibuprofen 400mg + oxycodone HCL 5mg was found to have the most effective analgesic effects in acute dental pain as measured by the objective efficacy measurements of SPID6 and TOTPAR6. It was reported in the literature that ibuprofen 400mg have a stronger analgesic efficacy than acetaminophen 1g [45, 46]. Post-operative inflammation may magnify the process of acute pain signals which potentially lead to greater pain nociception [47–50]. The anti-inflammatory action may therefore provide a higher analgesic efficacy. Moreover, oxycodone HCL is a stronger opioid when compared to codeine phosphate and hydrocodone bitartrate. According the equianalgesia chart [51, 52], the analgesic potency of oxycodone HCL is around 1.5–2.0 times stronger than hydrocodone bitartrate and 15–20 times stronger than codeine phosphate. The combination of the two analgesics therefore was found to be superior in terms of analgesic efficacy when compared to the other combinations.

Acetaminophen and codeine phosphate combination is a common analgesic combination in clinical practice. Acetaminophen mechanism of action is not fully understood [3]. It was suggested that the mechanism of acetaminophen may be related to inhibition of the nitric oxide synthase [51], reduction of spinal prostaglandin E2 release [53], or reversal of the hyperalgesia induced by N-methyl-D-aspartate (NMDA) [54, 55]. The side-effects of acetaminophen are minimal. Unlike NSAIDs, acetaminophen is not likely to cause gastrointestinal irritation. The prevalence of allergic reaction to acetaminophen is rarer when compared to the NSAIDs counterpart. We noted the efficacy of acetaminophen 600mg + codeine phosphate 60mg was doubled when compared to acetaminophen 300mg + codeine phosphate 30mg. We therefore concluded for post-operative pain control after third molar surgery, a higher dosage of acetaminophen and codeine phosphate combination would be better in terms of analgesic efficacy. However, it was also noted the raised dosage of codeine phosphate was related to an elevated prevalence of drowsiness, which might not favorable especially to drivers or machine operators. Taking all these factors into considerations, we recommend the combination of acetaminophen 600mg and codeine phosphate 60mg is effective for post-operative pain after third molar surgery, and may be useful when the patient is allergic to NSAIDs.

We noted most of the adverse effects of the combined analgesics from the included studies were mostly contributed by centrally acting analgesic i.e. codeine phosphate, hydrocodone bitartrate or oxycodone HCL. Their common side effects include drowsiness, dizziness, headache and nausea and vomiting [56, 57]. Other possible adverse effects of opioids described in literature e.g. itching, dry mouth, flashes, sweating and chills were also reported in our study but in a low prevalence [3, 58]. Severe adverse reactions of opioids in patients including tolerance, dependence, confusion, hallucinations, delirium, hypothermia, bradycardia/tachycardia, orthostatic hypotension and urinary retention were not found in our study [56, 57]. We believed those uncommon adverse effects were more likely found in prolonged use of opioids in chronic pain patients [59–61]. It was therefore very unlikely that a short-course use of analgesic for acute dental post-operative pain would lead to these major adverse effects or severe complications. Clinicians have an important role to prescribe appropriate dosing such that patients could gain the analgesic effects with the least adverse effects. Under suitable dosage, central acting analgesic could be an effective and safe medication for the treatment of acute dental post-operative pain.

One of the randomized clinical trials included in the final review compared 3 different dosages of the combination of ibuprofen and caffeine. Caffeine is the central-nervous-system stimulant which is an antagonist of adenosine receptors in the brain [62]. High dose of caffeine may cause tolerance, insomnia, hallucination, reduced control of fine motor movements [52, 63–65]. We reported the adverse effects of the combinations of ibuprofen and caffeine was minimal when compared to other combinations containing opioids. We also found the analgesic efficacies of ibuprofen 200mg with caffeine 100mg or 200mg were similar, with both much superior than with caffeine 50mg. We presumed that the caffeine ceiling dose may be approximately at around 100mg and the combination of ibuprofen 200mg with caffeine 200mg might not have an extra benefit in its analgesic effect.

The limitations of this systematic review and meta-analysis included the possibilities of reporting bias. Some pharmacological studies were sponsored by pharmaceutical companies, which might only report favourable outcomes if a drug combination was shown to be superior. Furthermore, our group did not request centers and companies to report if they had unpublished data on this topic, which might not find all related studies or data about the clinical question we defined.

In this study, the commonly reported adverse effects of NSAIDs (e.g. dyspepsia, gastric ulceration/bleeding, diarrhea) were not found in the drug combinations. Non-selective NSAIDs inhibit both cyclooxygenases (COX): COX-1 and COX-2 which reduce the levels of protective prostaglandins, leading to increase in gastric acid secretion and diminish bicarbonate secretion and mucus secretion [66, 67]. The included studies reported the use of analgesic combinations containing NSAIDs only in a very short course for the acute dental pain. The dosages and the duration of taking NSAIDs might not be sufficient to induce an obvious adverse effect in most patients. The introduction of COX-2 selective inhibitors was reported to have a strong analgesic effect with less adverse effect on the gastrointestinal tract when compared to the non-selective NSAIDs. Stichtenoth DO and Frölich JC have suggested that selective COX-2 inhibitors have significantly less gastric events and no effects on platelet aggregation [68]. However, a COX 2 selective inhibitor was found to increase cardiovascular risks and was withdrawn from the market [69, 70]. There are several COX-2 selective inhibitors still in the market and are found to be safe to use. In this study, there was no well conducted RCT on the efficacy and safety of analgesic combinations with a COX-2 selective inhibitor included in the final review. We therefore recommend future research to investigate the efficacy and side effects on the combination of COX-2 to another group of analgesic, which may potentially be a good analgesic choice for post-operative pain after third molar surgery.

## Conclusion

This systematic review and meta-analysis of randomized clinical trials has presented the efficacy and adverse effects of the various analgesic combinations for acute post-operative dental pain control. We have identified ibuprofen 400mg with oxycodone 5mg was more effective when compared to the other 16 combinations. Nausea was the most common adverse effects in an analgesic combination containing an opioid. Ibuprofen 200mg with caffeine 100mg or 200mg has a reasonable analgesic effect with fewer side effects when compared to the other analgesic combinations.

## Supporting Information

S1 Fig PRISMA checklist. (TIFF)

S2 Fig PRISMA checklist. (TIFF)
