# Supplementary figures and images for: The Efficacy and Clinical Safety of Various Analgesic Combinations for Post-Operative Pain after Third Molar Surgery: A Systematic Review and Meta-Analysis
Source: PLoS One. 2015 Jun 8;10(6):e0127611. doi: 10.1371/journal.pone.0127611 (PMC4459961; doi:10.1371/journal.pone.0127611)

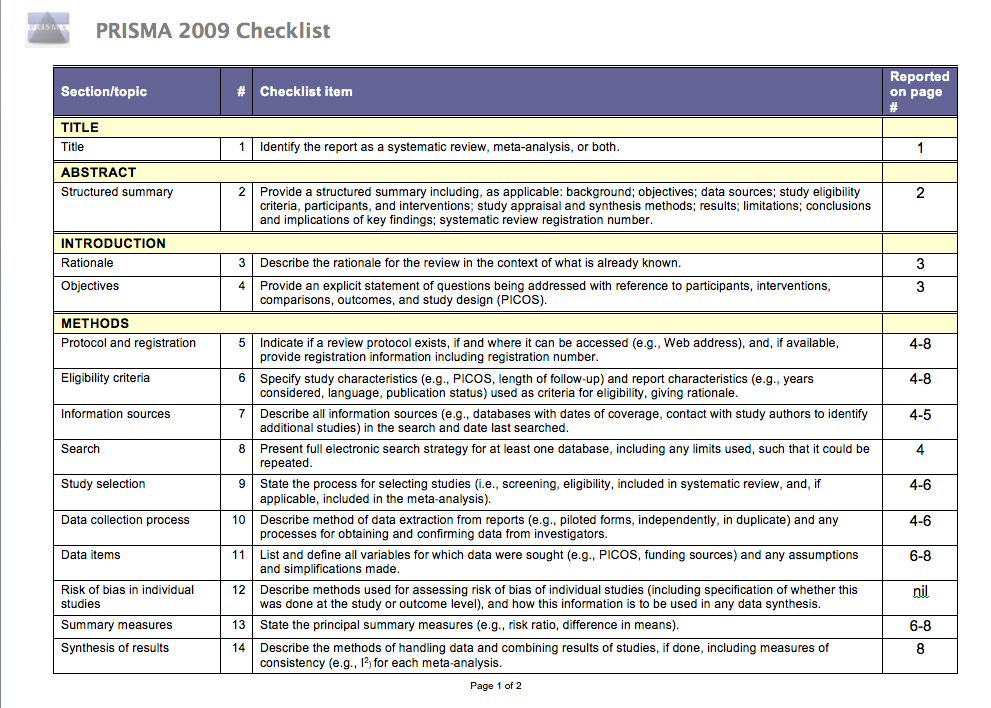

Supplement: S1 Fig — (TIFF) [file pone.0127611.s001.tiff]

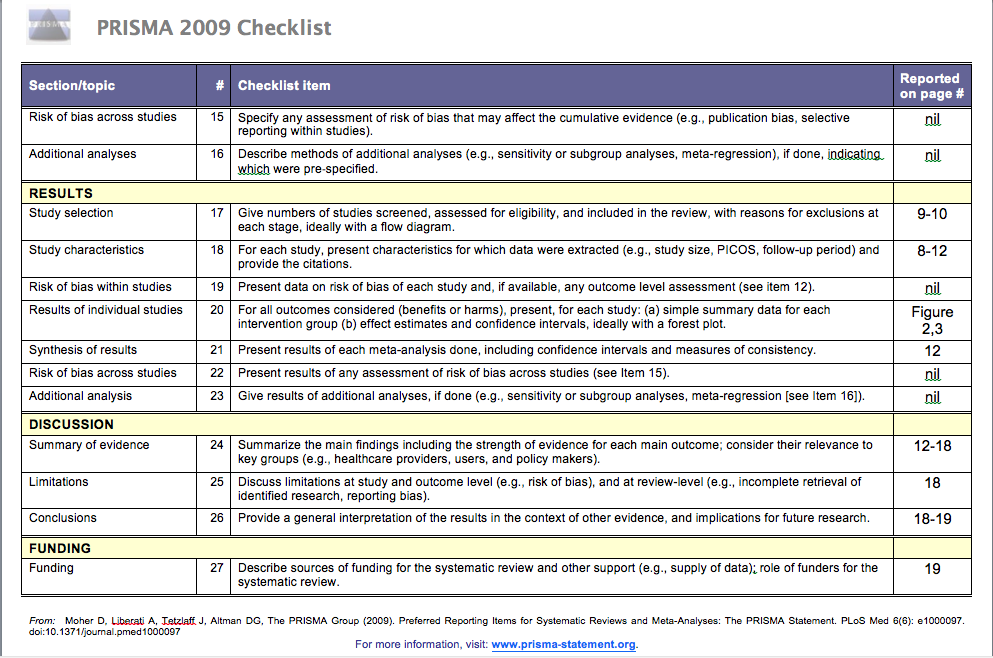

Supplement: S2 Fig — (TIFF) [file pone.0127611.s002.tiff]
